# Deep Brain Stimulation Frequency of the Subthalamic Nucleus Affects Phonemic and Action Fluency in Parkinson's Disease

**DOI:** 10.1155/2016/6760243

**Published:** 2016-12-05

**Authors:** Valéria de Carvalho Fagundes, Carlos R. M. Rieder, Aline Nunes da Cruz, Bárbara Costa Beber, Mirna Wetters Portuguez

**Affiliations:** ^1^Pontifical Catholic University of Rio Grande do Sul (PUCRS), Porto Alegre, RS, Brazil; ^2^Brain Institute of Rio Grande do Sul (InsCer), Porto Alegre, RS, Brazil; ^3^Hospital de Clínicas de Porto Alegre (HCPA), Porto Alegre, RS, Brazil; ^4^Federal University of Health Sciences from Porto Alegre (UFCSPA), Porto Alegre, RS, Brazil; ^5^Federal University of Rio Grande do Sul (UFRGS), Porto Alegre, RS, Brazil

## Abstract

*Introduction.* Deep brain stimulation of the subthalamic nucleus (STN-DBS) in Parkinson's disease (PD) has been linked to a decline in verbal fluency. The decline can be attributed to surgical effects, but the relative contributions of the stimulation parameters are not well understood. This study aimed to investigate the impact of the frequency of STN-DBS on the performance of verbal fluency tasks in patients with PD.* Methods.* Twenty individuals with PD who received bilateral STN-DBS were evaluated. Their performances of verbal fluency tasks (semantic, phonemic, action, and unconstrained fluencies) upon receiving low-frequency (60 Hz) and high-frequency (130 Hz) STN-DBS were assessed.* Results.* The performances of phonemic and action fluencies were significantly different between low- and high-frequency STN-DBS. Patients showed a decrease in these verbal fluencies for high-frequency STN-DBS.* Conclusion.* Low-frequency STN-DBS may be less harmful to the verbal fluency of PD patients.

## 1. Introduction

Deep brain stimulation (DBS) at the subthalamic nucleus (STN) improves motor function and the quality of life of patients with advanced Parkinson's disease (PD) [1]. However, some adverse effects are well documented in the literature, such as reduced verbal fluency (VF) [2–9].

The decline in VF observed in PD patients who undergo DBS is not well understood. Studies have hypothesized that this impairment is due to a possible lesion effect from surgery and/or an effect of the neurostimulator parameters, for instance, the frequency of stimulation [1, 2, 4, 6, 8, 9]. Thus, studies assessing the impact of neurostimulation parameters, such as stimulation frequency, on VF are needed.

Low-frequency stimulation has been associated with improved motor symptoms, including freezing of gait and swallowing, in patients with STN-DBS [10, 11]. Similarly, a study on the effects of low-frequency (10 Hz) and high-frequency (130 Hz) DBS-STN on semantic and phonemic verbal fluency found that performance on all VF tasks was significantly better for the low-frequency condition [12]. Further studies are needed to understand how frequency affects VF by analyzing additional VF tasks and the performance of various populations due to language and educational differences.

VF tasks are often used as operating measures of language and executive functions [13]. Among VF tasks are tasks that measure semantic VF (requests for words from a specific semantic group, such as animals or fruits) [13, 14], phonemic VF (requests for words that start with a certain letter) [13, 15], verb fluency or action fluency (requests for words designating things that people do) [13, 16], and unconstrained VF (requests any word without a criterion) [17].

The various VF tasks may provide different types of information regarding cognition because each VF task requires accessing specific lexical and/or semantic representations according to the criteria. The VF tasks activate overlapping areas of the frontal brain regions, but different word retrieval criteria likely activate additional distinct regions [13]. Semantic fluency is thought to be associated with temporal-lobe dysfunction, whereas phonemic fluency is associated with frontal-lobe dysfunction [18]. The action fluency deficit has been reported as a possible marker of frontostriatal impairment [16, 19]. Although both phonemic and action VF rely on frontal brain areas, evidence has indicated that action VF relies more heavily on semantic information that involves motor content (and because action VF may involve motor brain areas) [13, 19], whereas phonemic fluency relies more heavily on lexical retrieval to access words with phonemic similarities [13]. The action fluency task appears to be an important task for evaluating PD, as studies have shown that the action fluency task may be more sensitive to cognitive impairment in PD patients compared to other VF tasks [16, 19]. Currently, little is known regarding the neural and cognitive substrates of unconstrained VF because the few studies that have used this VF task only verified the influence of demographic factors on its performance [20, 21]. The absence of a retrieval criterion, as in the unconstrained VF task, may reinforce the need for inhibitory capacity, cognitive flexibility, working memory, and planning. Additionally, the unconstrained VF task may be considered as the absence of specific semantic or lexical involvement.

This study aimed to analyze the impact of low-frequency (60 Hz) and high-frequency (130 Hz) STN-DBS on VF tasks of PD patients. We hypothesized that PD patients would present VF deficits due to the frontosubcortical impairment caused by the disease. Thus, if there is an influence of the frequency of stimulation in our population, the frequency should affect the VF tasks that rely more on frontosubcortical functions, as required by the phonemic and action VFs. To test this hypothesis, not only is it important to assess phonemic and action VF tasks, but also the VF tasks that we hypothesized would not be affected by the frequency of stimulation, that is, semantic and unconstrained VFs.

## 2. Methods

### 2.1. Patients

The present study was a randomized double-blinded experimental study. The study was conducted with outpatients from the Neurology Service of* Hospital de Clínicas de Porto Alegre* (HCPA).

The study included 20 patients with idiopathic PD diagnosed according to the criteria of the UK Parkinson's Disease Society Brain Bank [22] and aged between 30 and 75 years. All participants had a bilateral STN-DBS implanted. Only patients with DBS parameters stabilized to the best motor control were included. Also, all patients were Brazilian Portuguese native speakers.

Exclusion criteria included the abuse of illicit drugs or benzodiazepines within the last six months, the presence of auditory impairment, as evaluated by an audiometric screening performed by an audiologist, the presence of visual impairment, a clinical diagnosis of depression or the presence of important signs or symptoms of depression (measured according to the 17-item Hamilton Depression Rating Scale, with a cutoff of 23 for very severe depression) [23], a clinical diagnosis of dementia or Mini-Mental State Examination (MMSE) with scores lower than the expected for the patient's educational level (cutoff of 20 for illiterates, 25 for 1 to 4 years of education, 26.5 for 5 to 8 years, 28 for 9 to 11 years, and 29 for higher levels of education) [24], a history of psychotic symptoms, or a history of alcoholism (screening according to the CAGE questionnaire with a score ≤1) [25].

### 2.2. Procedures

This study was conducted in a randomized, double-blinded manner. The order of the initial DBS conditions was defined by a medical student using the website http://www.random.com/. The order offered by the website was random but with an equal distribution of the initial DBS conditions. The low-frequency (60 Hz) and high-frequency (130 Hz) conditions were termed A-condition and B-condition, respectively. Each participant was assigned the AB order (*n* = 10) or the BA order (*n* = 10). A neurologist adjusted the frequency of stimulation, according to the randomization order, but was not allowed to participate in any rating or evaluation. The participants, the neuropsychologist who administered the VF tasks, and the neurologist who rated the Unified Parkinson's Disease Rating Scale (UPDRS-III) were blinded to the DBS condition. When the experiment was finalized, the A and B codes were revealed to compute the scores in the database.

After adjusting the frequency, the participants waited one hour to carry out the VF tasks. They then performed the following VF tasks: phonemic VF (FAS version and letter P version), semantic VF (animals), unconstrained VF, and action fluency. For the FAS version of the phonemic VF task, the participants were asked to say words beginning with the letters “F,” “A,” and “S” for one minute for each letter. The final score was the total number of words beginning with “F,” “A,” or “S” that the participants were able to say [12]. For the letter P version of the phonemic VF task, the participants were asked to say as many words as possible beginning with the letter “P” within two minutes [15, 26]. For the semantic VF task, the participants were asked to say as many animals as possible within one minute [14]. For the unconstrained VF, the participants were asked to say as many words as possible, excluding names and numbers, within 2.5 minutes while keeping their eyes closed [26]. For the action fluency task, the participants were asked to say as many actions or “things that people can do” as possible within one minute [16, 27]. The instruction of these previous VF did not allow participants to say proper names or numbers. On semantic VF, no score was given for subcategory (e.g., bird) if specific exemplars were also given (e.g., dove, canary). Additionally, sex- and age-specific names of the same animal species were considered to be the same animal (e.g., hen, rooster). On action VF, it was not allowed to use the same verb with different endings (e.g., eat, eating, eaten). Intrusions and perseverations were not scored [14–16, 28]. The UPDRS-III was used for the motor assessment [29]. After performing these evaluations, the neurologist readjusted the frequency of stimulation according to the assigned randomization to evaluate the other conditions. The participants waited one hour to repeat the VF testing and motor assessment. Upon completion of the assessments, the neurologist adjusted the parameters of the STN-DBS implanted back to the stabilized values used by each participant.

Demographic (sex, age, and education), cognitive (MMSE), and clinical (time of disease in years, time after surgery in months, Hamilton Depression Rating Scale, and the levodopa-equivalent dose (LED)) variables were considered in secondary analyses. The LED was measured as mg/day and was calculated using conversion formulae [30].

The ethics committees of out institution approved this study, and all participants gave written informed consent.

### 2.3. Statistical Analysis

Statistical analyses were performed using the Statistical Package for Social Sciences (SPSS version 21.0) with a significance level of 5% (*p* ≤ 0.05). Continuous variables were reported as the mean (M) and standard deviation (SD). Categorical variables were described by the absolute and relative frequencies. The distribution of variables was verified using the Shapiro-Wilk test. To compare the VF performance between 60 Hz and 130 Hz frequencies, we used the generalized estimating equation (GEE) model. To verify if any demographic, clinical, or cognitive aspects influenced the effect of stimulation frequency on VF, we conducted Spearman's correlation. We used the delta value of each VF task (VF task score for the 60 Hz condition minus the VF task score for the 130 Hz condition) and of the UPDRS-III scores (UPDRS-III score for 60 Hz minus UPDRS-III score for 130 Hz) in the correlational analysis.

## 3. Results

### 3.1. Patients Characteristics

The initial study sample consisted of 25 individuals; however, 5 were excluded for not meeting the inclusion criteria. Three included patients were not able to complete some of the verbal fluency tasks in both frequency conditions. However, these participants were still included in the data analysis. All participants were on levodopa drugs, only 6 participants were on amantadine (mean dose of 283.33 mg/day), and no one was on anticholinergics or antipsychotics. The baseline characteristics of the participants are presented in Table 1.  Table 2 presents sex, age, the parameters of stimulation, and the VF outcomes of the VF tasks that were significantly different between frequencies of stimulations, for each participant.

### 3.2. Verification of Practice Effect

The first and second sets of VF tasks were compared to determine if there was a practice/learning effect due to the repetition of the tasks. The results showed that there was no significant difference between the two sets of tasks for any of the VF tasks (Table 3).

### 3.3. Verification of the Effect of Stimulation Frequency

Table 4 shows that, after 60 Hz stimulation, the performances of the phonemic (FAS and P version) and action fluency tasks were significantly better than those after 130 Hz stimulation.

**Figure 1:**
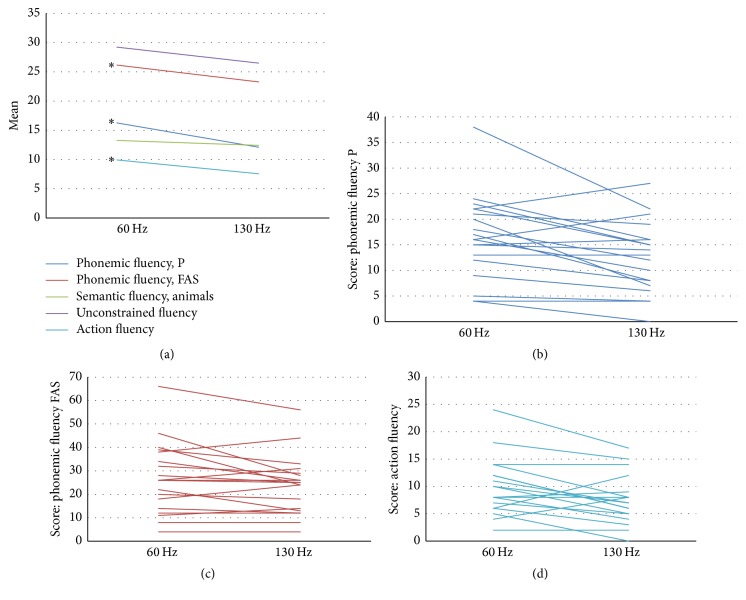
Patients' performances on VF tasks at low and high stimulation frequencies. (a) Performance of the entire sample for each VF task. *∗* denotes the significant difference between frequency conditions for the respective task. (b) Phonemic fluency performance (P version) by the patient. (c) Phonemic fluency performance (FAS version) by the patient. (d) Action fluency performance by the patient.

The performances for the VF tasks according to the STN-DBS frequency are shown in Figure 1(a). Despite the significant difference between stimulation conditions for phonemic and action fluencies, the individual performances for each of the VF tasks presented in Figures 1(b), 1(c), and 1(d) indicate that the participants did not exhibit the same outcome pattern. In addition to the comparison analyses, we assessed the distribution of patients who improved or worsened for 60 Hz stimulation in the phonemic and action fluency tasks. The differences (delta values) in the outcomes of the VF tasks between the frequency conditions (60 Hz minus 130 Hz conditions) were classified as “improvement at 60 Hz” (positive delta values), “worsening at 60 Hz” (negative values of delta), or “no difference” (zero values of delta) (Table 5). The individual description of the VF outcomes is also shown in Table 2.

### 3.4. Correlational Analysis

In the correlational analysis, we included the delta values of the VF task outcomes that showed significant differences between the frequency conditions (P and FAS versions of the phonemic fluency tasks and the action fluency task) to determine if the difference was correlated with additional variables (including the delta values of UPDRS-III). The delta value of the FAS version of the phonemic VF task was negatively associated with age (*r* = −0.0473; *p* = 0.041). The delta value of the P version of the phonemic VF task was negatively associated with UPDRS-III (*r* = −0.686; *p* = 0.002) (Table 6).

## 4. Discussion

The present study aimed to investigate the impact of modulating the frequency of STN-DBS on the performance of VF tasks in patients with PD. We assessed the effect of low-frequency stimulation of 60 Hz compared to high-frequency stimulation of 130 Hz in patients who had undergone bilateral STN-DBS in the medication-on state. We found that low-frequency stimulation had a positive impact on phonemic and action fluency, and this effect was not due to practice. Furthermore, we observed different outcome patterns for the VF tasks based on the frequency conditions, which could not be explained by the demographic, cognitive, and clinical variables that were studied here.

Previous studies have pointed to a decline in VF after STN-DBS surgery in PD patients, although the reason behind this decline is not well understood. There are many methodological differences among such studies, such as evaluations performed while stimulation is “on” or “off,” at pre- and postsurgical time points, and with or without a control group [2–9].

Greater declines in VF over time in STN-DBS patients compared to nonsurgical PD patients have been reported, which suggests that VF impairment is related to the DBS intervention [3]. VF may decline as a consequence of microsurgical injuries, which affect the cortical-basal circuits involved in the recovery process of words [31–33]. The number of microelectrode recordings required during surgery for lead placement does not adversely affect VF [4], although this finding does not exclude the possibility of an effect due to the lesions caused by the macroelectrode, suggesting that other factors in addition to microlesions may be involved in VF impairment after surgery.

The frequency of stimulation for treatment of PD has been studied in other clinical situations regarding STN-DBS. For example, 60 Hz stimulation, compared with the routine 130 Hz, improved swallowing function and freezing of gait in patients with PD who underwent bilateral STN-DBS [10, 11]. A previous study that evaluated semantic and phonemic VF for 10 Hz and 130 Hz stimulation reported greater performances in both VF tasks for low-frequency stimulation [12]. Our results also showed that low-frequency stimulation was associated with better scores of phonemic VF but no improvement was found for semantic VF. Wojtecki et al. [12] did not exclude participants with dementia or lower scores in their cognitive screening, and they did not describe the global cognitive status of the participants. It is known that patients with dementia may also present deficits in semantic VF [16]. Furthermore, differences in language and education may contribute to differences among populations.

When we compared the VF scores and the motor performances between the low-frequency (60 Hz) and high-frequency (130 Hz) stimulation trials, we found that phonemic and action fluency significantly declined for 130 Hz stimulation. However, no significant difference was found between stimulation frequencies for the semantic and unconstrained VF tasks. Our findings indicate that the influence of the stimulation frequency relies more heavily on specific frontosubcortical pathways involved in lexical-word and action-semantic processes, as there was an influence of frequency on phonemic and action VF tasks but not on semantic and unconstrained VF tasks. As we expected, the frequency affected the VF tasks that rely more heavily on frontosubcortical functions, which are impaired in PD, supporting our* a priori* hypothesis.

Phonemic and action fluency, which were hampered by high-frequency neurostimulation, are both tasks that involve frontal circuits and that rely more heavily on executive functions. Semantic VF depends on lexical-semantic processes and temporal circuits [13, 34]. Unconstrained VF is used to assess clinical conditions due to right or left hemisphere lesions, although there are no studies regarding its construct validity and the brain areas involved in adults [17]. One hypothesis regarding the cognitive processing of verbs, based on the theory of embodied cognition, states that the same brain areas involved in planning and motor execution participate in accessing lexical and semantic processes of verbs. This theory, though still controversial, helps to explain the deficits in verb production that have been observed in different clinical groups with Parkinsonian syndromes [35, 36]. STN-DBS could affect lexical-semantic processing of actions, such as those involved in the action fluency task; in the same way, it affects neural motor circuitry. The mechanism whereby the stimulus frequency may affect different circuitries remains unknown.

In the present study, no significant difference was found in the motor performance of the patients in relation to the stimulation frequency. One hypothesis for this finding is that the patients were under the effect of dopaminergic medication during the evaluation. If there was no effect of the medication, the low-frequency stimulation (60 Hz) would be expected to correspond to worse motor symptoms, whereas an improvement in motor performance for the high-frequency stimulation would be expected [10].

The comparison analyses revealed that most patients performed better in phonemic and action VF tasks for the low-frequency condition. However, a few of the participants presented an opposite pattern (worse VF scores for low-frequency stimulation) or no difference between the conditions. In an attempt to elucidate the cause of these different patterns, correlational analyses were conducted with the delta values of the phonemic and action VF scores. The scores from the FAS version of the phonemic VF task were negatively correlated with age, indicating a possible effect of age on the benefit of low-frequency stimulation; that is, older participants may exhibit lower improvements in performance for low-frequency stimulation compared to younger patients. The scores from the P version of the phonemic VF task were negatively correlated with UPDRS-III total score, indicating that increased improvements in motor performance were associated with smaller improvements in this phonemic VF task for low-frequency stimulation. This latter finding indicates that motor performance and phonemic VF scores (P version) are characterized by opposite outcomes at low-frequency stimulation. Future studies should seek identifying the factors that explain the different VF improvement profiles for varying frequency stimulation conditions by studying larger samples of PD patients with STN-DBS.

Our study is the first one that called attention to different outcomes of verbal fluency when frequency conditions were compared. Many aspects may influence the modulation of frequency on verbal fluency. Because the STN is thought to have separate functional subregions [37], we hypothesize that the volume and locus of activated STN tissue may interact with the effect of DBS frequency. Besides that, there are studies suggesting that DBS leads to neural plasticity in motor cortex and in modulating corticobasal circuits [38, 39]. Then, it is possible that frequency of stimulation may also interact with the effect of neural plasticity leading to different outcomes on verbal fluency. These possibilities of interactions may be further investigated in future studies.

Our results should be interpreted in light of some limitations. First, the improvement in the VF task for the low-frequency condition could be due to an improvement in other cognitive functions, such as attention. However, this study did not evaluate other cognitive functions. We chose to utilize a less-extensive assessment because some patients do not tolerate adjustments in stimulation frequency for long periods of time. Second, the participants were not evaluated in the DBS-off condition so that they were not exposed to unpleasant symptoms for long periods of time, and this study did not include a control group. The lack of information for the DBS-off condition and the lack of a control group do not allow surgical effects to be assessed. Third, the administration order of the VF tasks was the same in both conditions. There is the possibility of an order effect, but based on a previous study, we do not believe an order effect occurred, at least pertaining to the action VF task [27].

## 5. Conclusion

In summary, the results of the present study led to two important conclusions. First, the frequency of STN-DBS affects phonemic and action fluency in PD patients. Second, low-frequency (60 Hz) stimulation is associated with less negative side effects on VF than high-frequency (130 Hz) stimulation. Therefore, whenever possible, low-frequency stimulation should be the first choice for PD patients, especially for patients who present any cognitive impairments, such as reduced VF, in their daily activities. Future studies utilizing larger sample populations and those incorporating longer study periods should be performed to investigate stimulation effects on VF with regard to electrode position in the STN and other stimulation parameters (amplitude and pulse width).

## Figures and Tables

**Table 1:** Baseline characteristics of the participants.

Variables	Mean ± SD or *n* (%)	Range
Sex, male	16 (80)	—
Age	56.65 ± 10.71	31–75
Education	10.10 ± 5.23	2–22
Time of disease, years	15.30 ± 4.71	10–29
Levodopa equivalent dose, mg/day	1165.00 ± 615.08	300–2300
Time after surgery, months	2.21 ± 1.38	0–7
MMSE	26.45 ± 2.52	21–30
Hamilton Depression Rating Scale		
Normal	14 (70)	—
Mild depression	4 (20)	—
Moderate depression	2 (10)	—
Amplitude (V), left	3.02 (0.65)	1–3.90
Amplitude (V), right	2.98 (0.60)	1.80–3.60
Pulse width (*μ*s), left	79.50 (17.61)	60–120
Pulse width (*μ*s), right	81.00 (17.14)	60–120
Frequency (Hz), left and right	124.00 (26.04)	60–180

SD: standard deviation; MMSE: Mini-Mental State Examination.

**Table 2:** Stimulation parameters at the moment of inclusion and verbal fluency outcomes of each subject.

Subject	Sex	Age	Frequency (Hz)	Stimulation contacts (all cases +)		Amplitude (V)		Pulse width (*μ*s)		Verbal fluency outcomes (at 60 Hz)		
				L	R	L	R	L	R	Action	Phonemic (P)	Phonemic (FAS)
1	M	49	90	3-	11-	3.0	3.0	60	60	—	↑	↑
2	F	66	110	3-	10-	3.6	1.8	90	90	↓	↑	↑
3	M	75	130	0-, 1-	8-, 9-	3.3	3.3	60	60	ND	↑	ND
4	M	65	130	2-, 3-	11-	3.0	3.6	60	90	↑	↑	↓
5	F	62	110	2-	11-	2.8	2.6	60	60	ND	↓	ND
6	M	53	180	1-	9-, 10-	2.6	2.7	90	90	↑	↑	↓
7	M	31	140	2-, 3-	8-, 9-	3.6	3.6	90	120	↑	↑	↑
8	M	50	60	0-	8-, 9-	2.5	3.0	90	90	↑	↑	↑
9	M	70	130	1-, 2-	9-, 10-	3.0	3.2	90	90	↑	ND	↑
10	M	47	110	0-	11-	2.8	2.8	60	60	↓	↑	↑
11	M	46	140	0-	9-	2.6	3.0	90	90	↑	↑	↑
12	M	70	110	3-	11-	3.6	3.6	90	90	ND	↑	↑
13	M	59	160	2-	8-	3.5	3.5	120	90	↑	↑	ND
14	M	47	110	1-	9-	1.0	2.0	60	90	—	—	—
15	M	61	120	2-	11-	3.4	3.4	60	60	↓	↑	↑
16	M	66	160	2-	9-	3.2	3.6	90	90	↑	ND	ND
17	F	59	120	2-	5-	3.2	3.2	90	90	↑	↑	↑
18	F	48	130	3-	8-	3.9	2.2	60	60	—	—	—
19	M	51	110	2-	10-	3.6	3.6	90	90	↑	↑	↑
20	M	58	130	0-	8-	2.2	2.0	90	60	—	↓	↓

F: female; L: left; M: male; R: right; ↑: improvement; ↓: worsening; ND: no difference; “-”: cathode/negative electrode contact.

**Table 3:** Comparisons of verbal fluency tasks between moments of administration.

Verbal fluency task	Moment 1	Moment 2	Difference	CI 95%	*p*
	Mean ± SD	Mean ± SD			
Phonemic, P	14.53 ± 1.61	14.47 ± 1.89	0.05	−2.96–3.07	0.973
Phonemic, FAS	25.26 ± 2.88	25.39 ± 15.33	−0.11	−3.36–3.15	0.949
Semantic, animals	13.26 ± 1.07	13.42 ± 1.22	−0.16	−1.86–1.54	0.856
Unconstrained	29.63 ± 2.50	27.58 ± 3.00	2.05	−1.76–5.87	0.292
Action	8.39 ± 1.00	9.11 ± 1.30	−0.72	−2.65–1.21	0.463

SD: standard deviation; CI: confidence interval.

**Table 4:** Comparisons of the verbal fluency tasks and UPDRS-III between the different frequencies of SNT-DBS.

Variables	60 Hz	130 Hz	Difference	CI 95%	*p*
	Mean ± SE	Mean ± SE			
UPDRS III, total	34.33 ± 4.74	35.44 ± 4.30	−1.11	−9.38–7.15	0.792
UPDRS III, tremor	2.72 ± 1.20	2.00 ± 1.09	0.72	−2.19–3.63	0.627
UPDRS III, gait	1.28 ± 0.26	1.61 ± 0.33	−0.33	−0.80–0.13	0.157
UPDRS III, pull test	1.28 ± 0.29	1.83 ± 0.34	−0.56	−1.12–0.00	0.052
Phonemic VF, P	16.53 ± 1.82	12.47 ± 1.56	4.05	1.65–6.45	0.001^*∗*^
Phonemic VF, FAS	26.84 ± 3.36	23.79 ± 2.79	3.05	0.10–6.00	0.042^*∗*^
Semantic VF, animals	13.70 ± 1.20	12.89 ± 1.08	0.89	−0.76–2.55	0.290
Unconstrained VF	29.63 ± 2.92	27.58 ± 2.59	1.95	−1.76–5.87	0.292
Action VF	9.94 ± 1.22	7.56 ± 1.94	2.39	0.77–4.00	0.004^*∗*^

CI: confidence interval; SE: standard error; UPDRS: Unified Parkinson's Disease Rating Scale; VF: verbal fluency; ^*∗*^ *p* ≤ 0.05.

**Table 5:** Distribution of delta values for phonemic and action fluency tasks.

	Phonemic VF	Phonemic VF	Action VF
	P version	FAS version	
Delta value, mean ± SD	4.05 (5.50)	3.05 (6.73)	2.40 (3.60)
Delta classification, *n* (%)			
Improvement at 60 Hz	14 (70)	11 (55)	10 (50)
Worsening at 60 Hz	2 (10)	3 (15)	3 (15)
No difference	2 (10)	4 (20)	3 (15)

SD: standard deviation; VF: verbal fluency.

**Table 6:** Correlational analysis between VF tasks (phonemic and action), demographic, cognitive, and clinical measures.

	Phonemic - P		Phonemic - FAS		Action	
	*r*	*p*	*r*	*p*	*r*	*p*
Age	−0.207	0.382	−0.473	0.041^*∗*^	−0.352	0.152
Education	−0.192	0.416	0.300	0.212	−0.021	0.935
MMSE	−0.014	0.953	0.215	0.377	0.108	0.669
Time of disease, years	0.400	0.081	0.358	0.132	−0.036	0.887
Time after surgery, months	0.288	0.218	0.440	0.060	0.284	0.253
Levodopa equivalent dose	0.351	0.140	0.131	0.592	−0.003	0.992
HDRS, total score	0.011	0.964	0.078	0.757	−0.138	0.599
UPDRS, total	−0.686	0.002^*∗*^	−0.342	0.165	−0.058	0.825
UPDRS, tremor	−0.200	0.426	−0.133	0.600	0.020	0.939
UPDRS, gait	−0.378	0.122	0.004	0.986	0.003	0.990
UPDRS, pull test	−0.312	0.207	−0.479	0.068	−0.321	0.209

HDRS: Hamilton Depression Rating Scale; MMSE: Mini-Mental State Examination; UPDRS: Unified Parkinson's Disease Rating Scale; ^*∗*^ *p* ≤ 0.05.
